# Changes in Fatty Acid Composition and Distribution of N-3 Fatty Acids in Goat Tissues Fed Different Levels of Whole Linseed

**DOI:** 10.1155/2014/934154

**Published:** 2014-11-11

**Authors:** Kamaleldin Abuelfatah, Md. Zuki Abu Bakar Zakaria, Goh Yong Meng, Awis Qurni Sazili

**Affiliations:** ^1^Faculty of Veterinary Medicine, Universiti Putra Malaysia (UPM), 43400 Serdang, Selangor, Malaysia; ^2^Faculty of Animal Production, University of Khartoum, Shambat, 13314 Khartoum North, Sudan; ^3^Institute of Biosciences, Universiti Putra Malaysia (UPM), 43400 Serdang, Selangor, Malaysia; ^4^Institute of Tropical Agriculture, Universiti Putra Malaysia (UPM), 43400 Serdang, Selangor, Malaysia; ^5^Department of Animal Science, Universiti Putra Malaysia (UPM), 43400 Serdang, Selangor, Malaysia

## Abstract

The effects of feeding different levels of whole linseed on fatty acid (FA) composition of muscles and adipose tissues of goat were investigated. Twenty-four Crossed Boer bucks were assigned randomly into three treatment diets: L0, L10, or L20, containing 0%, 10%, or 20% whole linseed, respectively. The goats were slaughtered after 110 days of feeding. Samples from the *longissimus dorsi*, *supraspinatus*, *semitendinosus*, and subcutaneous fat (SF) and perirenal fat (PF) were taken for FA analyses. In muscles, the average increments in *α*-linolenic (ALA) and total n-3 PUFA were 6.48 and 3.4, and 11.48 and 4.78 for L10 and L20, respectively. In the adipose tissues, the increments in ALA and total n-3 PUFA were 3.07- and 6.92-fold and 3.00- and 7.54-fold in SF and PF for L10 and L20, respectively. The n-6 : n-3 ratio of the muscles was decreased from up to 8.86 in L0 to 2 or less in L10 and L20. The PUFA : SFA ratio was increased in all the tissues of L20 compared to L0. It is concluded that both inclusion levels (10% and 20%) of whole linseed in goat diets resulted in producing meat highly enriched with n-3 PUFA with desirable n-6 : n-3 ratio.

## 1. Introduction

The increased intake of n-3 polyunsaturated fatty acids (PUFA), specifically, eicosapentaenoic acid (EPA) and docosahexaenoic acid has been associated with significant physiological and health benefits in human populations. The intake of n-3 PUFA provided benefits for reducing of the incidence of cardiovascular diseases, atherosclerosis, hypertension [1], some cancer, inflammatory diseases [2], and some mental and emotional disorder [3], in addition to improving eye and brain development and learning ability [4]. The potential benefits of n-3 PUFA have stimulated the research in different fields in order to increase these beneficial fatty acids (FAs) in the human diet towards recommended levels. Red meat, particularly that from ruminant animals, has a bad reputation attributed to its high saturated nature, a low ratio of PUFA to saturated FA (SFA), and high n-6 : n-3 ratio [5–7], which may cause numerous cancers, atherosclerosis, and coronary heart diseases [8, 9].

In response to the concerns of the medical community and health-conscious consumers, research in meat production has focused on altering the FA content of meat through increasing the n-3 PUFA content, decreasing the PUFA n-6 : n-3 ratio, and enhancing the content of CLA in meat. Comparing to monogastrics, increasing the PUFA in the ruminant meat is more challenging, since most of the PUFA in the animal diet are hydrogenated by the rumen microorganisms [10]. However, the inclusion of sources of *α*-linolenic acid (ALA) in the diets of ruminants has been shown to increase the concentration of n-3 PUFA in their meat. Feeding entire oilseeds is one of the strategies to reduce rumen biohydrogenation, as the seed coat provides protection for unsaturated FA (UFA) from rumen microorganisms [11], and might have lesser adverse effects on rumen fermentation than feeding free oils [12]. In addition, using intact seeds instead of oils has practical advantages in terms of handling the feed ingredients and manufacturing. Linseed (*Linum usitatissimum*) is a leading source of plant based n-3 FA containing about 40% oil, of which 50–60% ALA [13].

During the last decades, goat meat has gained a growing interest due to its preferable nutritional features, as it contains low levels of fat and cholesterol [14] and higher level of PUFA compared to beef or lamb [15]. The naturally high level of PUFA may indicate that goat has a potential to deposit high level of n-3 PUFA in it tissues. Enriching goat meat with n-3 PUFA together with its natural favorable nutrition characteristics enables goat meat to play an important role in human health as functional food, especially for health-conscious consumers. However, information about the effects of the feeding dietary regime on the FA profiles of edible tissues of goat, generally, is relatively scare [16]. Worse still, there is no report about the effects of feeding whole linseed, particularly, on the FA profiles of edible tissues of goat. Therefore, the objective of this study was to investigate the effects of moderate and high inclusion levels of whole linseed, as a source of n-3 PUFA in diets, on the FA composition of intramuscular and adipose tissues, with an emphasis on n-3 PUFA and CLA, of Crossed Boer goats.

## 2. Material and Methods

### 2.1. Experimental Animals and Housing

The trial was conducted at the Experimental Ruminant Unit, Department of Animal Science, Faculty of Agriculture, Universiti Putra Malaysia (UPM), under a tropical climate. Twenty-four, 5-month old Crossed Boer bucks with initial body weight (mean and SE) of 14.23 ± 0.33 kg were unique numbering, dewormed, and housed in individual wooden pens and subjected to an adaptation period of three weeks prior to the beginning of the feeding trial to adjust to the housing conditions and diets. At the end of the adaptation period, the initial body weight of each goat was recorded. Accordingly, they were distributed into three groups of eight animals in each group, where the mean live weight of animals was not significantly different between the experimental groups. The animals were allocated randomly to one of three of experimental diets.

### 2.2. Feeds and Feeding

Three diets, L0, L10, and L20, containing 0%, 10%, or 20% of whole linseed, respectively, were formulated to meet the nutrient requirements of growing goats [17]. The high inclusion level of linseed was determined at 20% to give ether extract less than 10%, which considered the maximum level for ruminants' diet [18]. The experimental diets and their ingredients and chemical composition are given in Table 1. The diets were randomly allocated to the three animal groups. Throughout the feeding trial, animals were fed at 3% body weight as dry matter intake daily. The trial period lasted for 110 days. Samples for proximate analyses were taken from the feed and refusals on a weekly interval.

### 2.3. Slaughtering and Tissues Sampling

At the end of the experiment, animals were slaughtered according to Islamic Halal-method (slitting the throat to cut the jugular veins and arteries without stunning) at the slaughter house, Department of Animal Science, Faculty of Agriculture, UPM. Samples were taken from* longissimus dorsi *(LD),* supraspinatus *(SS), and* semitendinosus* (ST) muscles. Additionally, about 10 g of perirenal fat (PF) and subcutaneous fat (SF) were obtained. All the tissues were then vacuum packaged and stored at −80 until fatty acid analyses.

### 2.4. Chemical Analysis

Samples of feed were subjected to proximate analysis according to the standard methods of AOAC (2007) [19]. For determination of dry matter (DM) content, the samples were dried at 105°C for 24 h in a forced-air oven. Ash content was determined by combusting the samples in a muffle furnace at 550°C for 6 h, and organic matter (OM) content was calculated by difference (OM = 100-ash content). The N content of samples was determined using a Kjeltec Auto Analyzer (Tecator, Hoganas, Sweden), and crude protein (CF) was calculated as N × 6.25. Ether extract (EE) was determined in petroleum ether using a Soxtec Auto Analyzer (Tecator). Neutral detergent fiber (NDF) and acid detergent fiber were determined using the procedures of [20].

### 2.5. Measurement of Fatty Acids

The total FAs were extracted from feeds and animal tissues based on the method of [21] modified by [22], using chloroform-methanol 2 : 1 (v/v) containing butylated hydroxytoluene to prevent oxidation during sample preparation. FAs were transmethylated to their FA methyl esters (FAME) using 0.66 N KOH in 14% methanol and methanolic boron trifluoride (BF_3_) according to the methods by AOAC (2000). The FAME composition was quantified with a gas-liquid chromatography on an Agilent 7890A GS system (Agilent, Palo Alto, CA, USA) equipped with a 100 m × 0.25 mm ID (0.20 *μ*m film thickness) Supelco sp-2560 capillary column (Supelco, Inc., Bellefonte, PA, USA). One *μ*L of FAME was injected by an autosampler. H_2_ was used as the carrier gas and the split ratio was 10 : 1 after FAME injection. The injector and detector temperature were programmed at 250°C and 300°C, respectively. The column temperature program initiated at 120°C held for 5 min, increased by 2°C/min up to 170°C for 15 min, and then the temperature increased again by 5°C/min up to 200°C for 5 min and increased again by 2°C/min to a final temperature at 235°C and held for 10 min. A reference standard (mix C4–C24 methyl esters; Sigma-Aldrich, Inc., St. Louis, Mo, USA) and CLA standard mix (cis-9 trans-11 and trans-10 cis-12 CLA) (Sigma-Aldrich, Inc., St. Louis, Mo, USA) were used for determining individual FA.

### 2.6. Statistical Analysis

Results were analyzed using analysis of variance with the different inclusions of linseed as the main effects. For feed data sets one-way ANOVA was used to compare differences in overall and individual fatty acid types. The tissue FA datasets were first analyzed using the one-way ANOVA within tissue type for treatment effects. Following this, a two-way ANOVA was employed to analyze for the effect of tissue type (or anatomical location of muscles) X treatment effects. In both cases Duncan's multiple range test was employed to elucidate significant means using the SAS software package, version 9.2. Differences between the least squared means were considered to be significant at *P* < 0.05. Data were presented as least-square means ± standard errors.

## 3. Result

### 3.1. Fatty Acid Composition of the Diets

The FA profiles of the diets are shown in Table 2. The most abundant FAs in the diets were oleic (33.5–27.1%), linolenic acid (LA) (21.9–18.0%), palmitic (28.07–7.89%), and ALA (39.3–1.9%). The proportions of oleic and palmitic were higher (*P* < 0.01) in L0 compared to L10 and L20. There was no significant difference in the proportion of LA among the treatment diets. As projected, the inclusion of linseed contributed to a significant variation in the content of n-3 PUFA. The highest proportion of n-3 FA was in L20 (39.3% of total FA), followed by L10 (33.4% of total FA), and the lowest was in L0 (1.6% of total FA). The SFA was higher (*P* < 0.001) in L0 (42.5% of total FA) compared to both L10 (17.4% of total FA) and L20 (15.5% of total FA). There were no significant differences among the experimental diets in n-6 PUFA.

### 3.2. Fatty Acid Composition of Tissues

The FA profiles for different goat muscles (LD, SS, and ST) and adipose tissues (SF and PF) are shown in Tables 3–7, respectively. The values are expressed as a percentage of the total fatty acids. The most abundant FAs in the goat muscles lipid were oleic acid (32.40–39.45%), palmitic acid (16.41–20.94%), and stearic acid (15.09–16.85%). The proportion of palmitic acid was greater (*P* < 0.05) in all muscles of the control group (L0) compared to the other groups (L10 and L20). Nonsignificant difference was detected in the proportions of stearic acid and oleic acid in the studied muscles, except for ST of L20, which showed a lower level of oleic acid compared to the same muscle for both L0 and L20. Similar to the muscles, oleic, palmitic, and stearic acids were the major FA in the adipose tissues. In SF, the proportion of oleic acid ranged between 37.14 and 43.13%. The higher proportion was exhibited by L10 with a significant difference (*P* < 0.05) when compared to L0. In the PF, the proportion of oleic acid ranged between 23.66 and 25.08%, with no significant differences across treatments. Both the SF and PF of the control group had higher proportions of palmitic acid (*P* < 0.005). The stearic acid in the SF ranged between 21.44 and 26.76%, and the control group scored the highest proportion compared to the other groups (*P* < 0.05). In the PF, the stearic acid ranged between 33.14 and 37.93% with no difference between groups.

**Figure 1:**
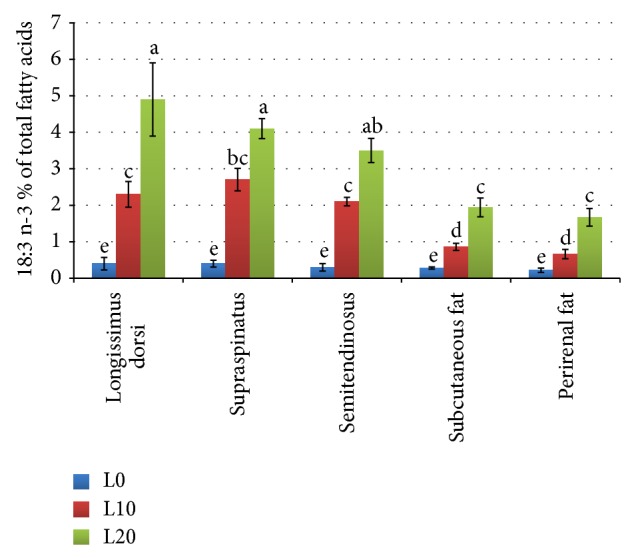
ALA (18 : 3 n-3) contents in muscles and fat tissues of goats fed diets containing different levels of whole linseed. Error bar = (1 SE). Bars with different alphabet notation differ significantly.

The ALA content in different tissues is shown in Figure 1. The proportion of ALA increased in all the studied tissues (*P* < 0.01) as the inclusion level of linseed increased. In muscles (Tables 3, 4, and 5), it ranged from 0.30% in SS of L0 to 4.85% in LD of L20. The highest proportion was exhibited by the LD muscle of goats fed the L20 diet. In the adipose tissues (Tables 6 and 7), the percentage of ALA was between 0.22% in the PF of L0 and 1.94% in the ST.

The increment of ALA in the different muscles of experimental groups was 5.7, 6.75, and 7.0 fold for L10 and was 12.52, 10.25, and 11.67 fold for L20 in LD, SS, and ST muscles, respectively, compared to L0. The increment of ALA in the adipose tissues was 3.07 and 6.92 fold and was 3.00 and 7.54 fold in the SF and PF for L10 and L20, respectively, compared to L0.

**Figure 2:**
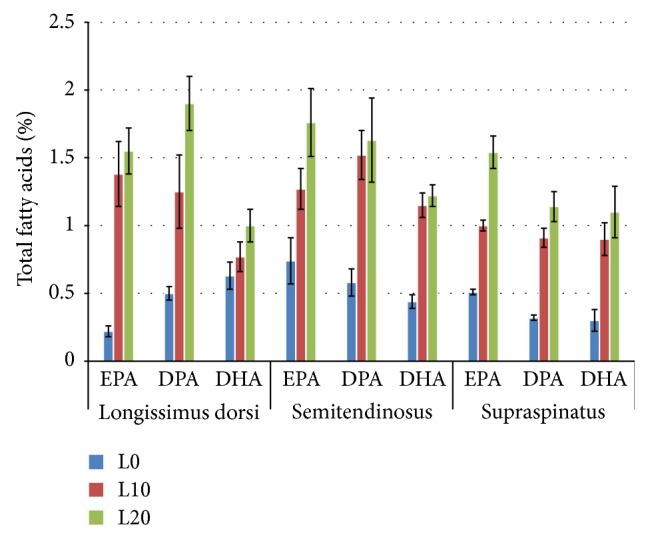
Long-chain n-3 FA contents in muscles and fat tissues of goats fed diets containing different levels of whole linseed. Error bar = (1 SE).

As illustrated in Figure 2, inclusion of the linseed in the goat diets also resulted in a significant increase (*P* < 0.05) in the long-chain n-3 PUFA, EPA (C20:5 n-3), DPA (C20:5 n-3), and DHA (C20:6 n-3). However, these long-chain n-3 FAs were not detected in the adipose tissues (SF and PF) of goats, where the ALA represented the entire n-3 PUFA detected in this tissue.

There were no significant differences in the proportions of LA and total n-6 in SS, ST, and SF; nevertheless they were higher in LD of control group and PF of L20.

The proportion of C18:1 trans-11 (vaccenic acid) was higher (*P* < 0.05) in the LD (1.99%), SS (1.51%), and SF (4.25%) of the L20 group compared to control group (1.44%, 1.65%, and 1.65%, for the same tissues, resp.).

**Figure 3:**
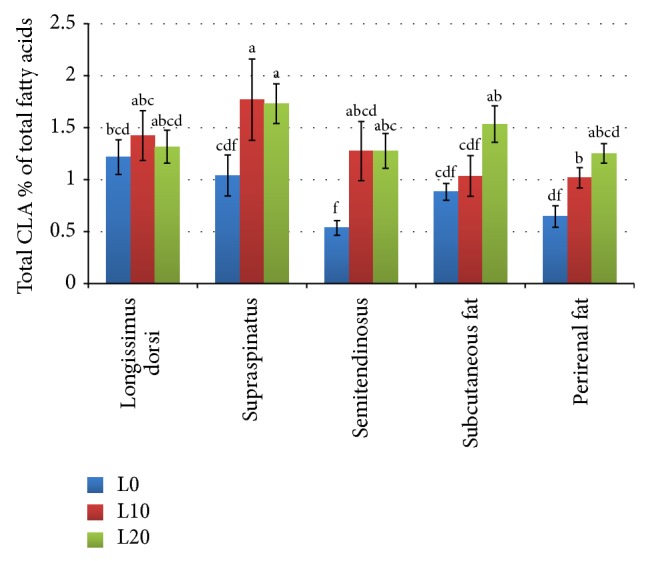
Total CLA contents in muscles and fat tissues of goats fed diets containing different levels of whole linseed. Error bar = (1 SE). Bars with different alphabet notation differ significantly.

As shown in Figure 3, the inclusion of linseed at 10% and 20% increased the total CLA in all the tissues compared to the control (*P* < 0.05), except for the LD muscle. The highest percentage (1.83) was found in the SS muscle of the L20 group, whereas the lowest percentage (0.54) was detected in the ST muscle of the control group (L0).

**Figure 4:**
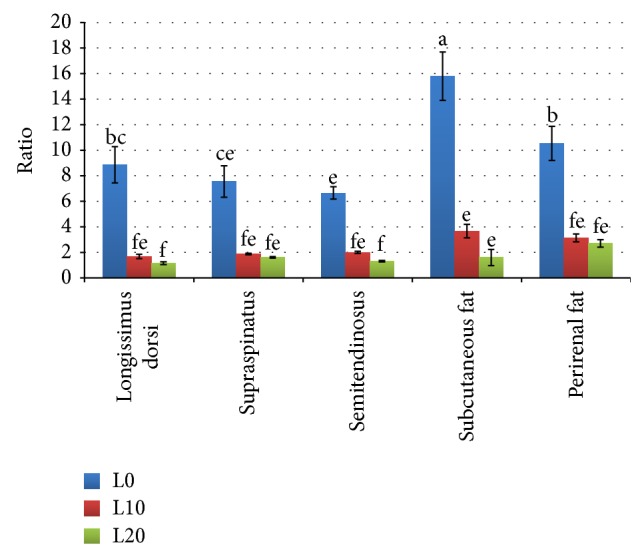
n-6 : n-3 ratio in muscles and fat tissues of goats fed diets containing different levels of whole linseed. Error bar = (1 SE). Bars with different alphabet notation differ significantly.

The PUFA n-6 : n-3 ratios for the tissues of goats fed different levels of linseed are summarized in Figure 4. The PUFA n-6 : n-3 ratios for the tissues of goats fed different levels of linseed are summarized in Figure 4 The highest ratio (15.79) was noticed in the SF of the control group (L0), while the lowest (1.16) was shown in the LD muscle of L20. Both inclusion levels of linseed (10% or 20%) dramatically reduced the n-6 : n-3 ratios of goat tissues (*P* < 0.001). In the muscle, the ratios decreased from 8.86, 7.55, and 6.65 in LD, SS, and ST, respectively, for the L0 group, to 1.68, 1.87, and 2.0 for L10 and to 1.16, 1.8, and 1.32 for L20, respectively, for the same muscles. Similar to the muscles, the n-6 : n-3 ratios of the SF and PF decreased from 15.79 and 10.53 in L0 to 3.67 and 3.14 for L10 and to 1.60 and 2.72 for L20, respectively. However, the difference between the groups fed linseed (L10 and L20) was not significant for all the tissues studied.

## 4. Discussion

The current paper is focusing on the effects of feeding different levels of whole linseed on FA profile with an emphasis on n-3 PUFA and CLA in different tissues of goat. The data related to growth performance and carcass characteristics of the experimental animal was reported in a previous paper by the same authors [23]. In general, the FA composition of meat can be greatly modified by diet [24]. Other factors, such as species, age, weight, sex, and breed, also influence the composition of FA. Additionally, the fatty acid composition differs between tissue sites in the animal body [25].

Similar to that reported in related studies [26, 27], the most abundant FAs in the experimental diets were oleic acid, LA. However, the level of ALA was intentionally higher in the L10 and L20, as a result of the inclusion of linseed. In tissues, the major FAs were oleic, palmitic, stearic, and LA, as previously reported in goat [15]; However, in this study the goats fed linseed diets showed a markedly high level of n-3 fatty acids in their tissues.

In this study, the levels of ALA and total n-3 PUFA in the muscles and adipose tissues were significantly increased in all goat tissues as the inclusion level of linseed increased in the diet. This result is in agreement with similar studies in sheep [28] and in beef [26, 29, 30]. Similar results were also reported in monogastric animals, for example, in pigs [31], in rabbits [32], and in chickens [33]. The average increments of ALA for the muscles were 6.48 and 11.48 fold for L10 and L20, respectively, compared to control group (L0). As we hypothesized, this finding was higher than previously reported in sheep by [28, 34] or in beef [35–43]. The higher increment of PUFA in goat muscles in current study can be attributed to a number of reasons. Firstly, the natural ability of goats to deposit PUFA is higher than that of cattle or sheep [15] since the species is considered to be one of the important factors affecting the FA composition in tissues [25, 44]. Secondly, the natural lower total lipids in goat meat compared to sheep or beef [14] play an important role in the high proportion of PUFA in goat meat, based on the findings of [44] in that “breeds or genetic types with a low concentration of total lipid in muscle, in which phospholipids are a high proportion of the total, will have higher proportions of PUFA in total lipid.” Thirdly, feeding whole seeds, which partially provides protection for the unsaturated FA from rumen microorganisms [11], collectively with the high rate of passage of feedstuffs through the rumen of goats compared to large ruminants [45], should also result in less extensive biohydrogenation of FA compared to large ruminants [46].

The increase in LC n-3 (EPA, DPA, and DHA), which are metabolic products of ALA, was higher than generally reported when feeding diets rich in ALA to sheep [47–49] or beef [26, 50, 51]. This increase in LC n-3 may indicate that the desaturation and elongation occur in goat muscle when fed linseed was sufficient to increase the synthesis of the active metabolites EPA and DPA, and even DHA, which did not show a significant increase in the previously mentioned studies on sheep or beef. [27] also reported a linear response in DHA to the concentration of ALA in goat fed diets containing low ratios of n-6 : n-3. However, similar findings were also reported by [52] in the polar lipids of lambs when they replaced sunflower oil with a linseed diet. Nevertheless, the supplementation of fish oil reported to be more efficient in increasing these beneficial FAs in the meat of ruminants [49] and monogastric animals [53]. Although, using fish oil may cause undesirable off-odor and flavor and color changes and decrease meat shelf life [44, 54, 55].

In this study, the proportion of C18:1 trans-11 (vaccenic acid) was significantly higher in the most tissues of animals fed 20% linseed. This finding is reasonable since vaccenic acid is an intermediate product of the microbial biohydrogenation of LA and ALA [56]. The increase of vaccenic acid in animal tissues is preferable since it performs as a precursor for the tissue biosynthesis of CLA [57] and may exert health benefits similar to those related to CLA in humans [58]. The increase in the total CLA in the most tissues goats fed linseed agrees with [59] who confirmed that varied rumen microorganisms are capable of the creation of numerous CLA and 18 : 3 isomers from ALA acid. However, different studies showed that safflower and sunflower seed, which are considered as a source of LA, are more efficient in increasing the concentration of CLA compared to linseed [60].

The lower proportion of total SFA in most tissues (SS, ST, and subcutaneous fat) of goats fed linseed (*P* < 0.05) is in disagreement with that reported in similar studies in sheep and beef. In this study, the reduction in total SFA is mainly due to the marked increase in the proportion of n-3 PUFA and the significant decrease in the proportion of palmitic acid. The decrease in the proportion of palmitic acid was also reported in young bulls fed linseed [61]. It is important to mention that palmitic acid was found to increase the plasma cholesterol level in human, while stearic does not exhibit such an effect [62, 63]; therefore, a high intake of palmitic acid increases the risk of atherosclerosis and cardiovascular diseases.

The increase in the PUFA: SFA ratios in all the muscles of the goats fed linseed, which ranged from 0.44 in LD of L10 up to 0.6 in SS of goats feed L20, can be mainly attributed to the increase in total n-3 PUFA since the proportion of n-6 PUFA was not affected by the treatment in most cases. Contrary, the increase in n-3 PUFA led to reduction in the n-6 : n-3 ratios, from values over 6.65 in the control group to values of 2.0 or less in the muscles of goats that were fed linseed. This result is similar to that reported in lambs fed different level of linseed [47] and higher than that observed in beef [26]. In general, the n-6 : n-3 ratio is highly affected by the type of unsaturated FA fed to the animals [46]. Although there was a similar decrease in n-6 : n-3 ratio in the adipose tissues form above 10 in the control to below 4, the PUFA:SFA ratio was very low (about 0.01) in these tissues and in this situation n-6 : n-3 ratio may give an indistinct indicator. Therefore, the absolute quantity of essential fatty acids consumed, rather than their n-6 : n-3 ratio, should be the first concern for the effective benefit from their intake [64].

## 5. Conclusion

The inclusion of linseed in the diet resulted in an increase in the proportion of ALA and total n-3 PUFA in muscles and adipose tissues of goats as the inclusion level of linseed increased. Furthermore, it increased the proportions of beneficial long-chains n-3 PUFA EPA, DPA, and DHA in the muscles. The inclusion of linseed at the 10% or 20% level can successfully improve the PUFA : SFA and n-6 : n-3 ratios to that recommended by the international health organization. Additionally, feeding linseed resulted in an increase in the total CLA, especially in the subcutaneous fat, and a decrease in the proportion of palmitic acid in all tissues.

## Figures and Tables

**Table 1:** Formulation and proximate analyses of the experimental diets.

Ingredient, % dry matter	Experimental diets^1^		
	L0	L10	L20
Whole linseed	—	10	20
Palm kernel cake	40	30	20
Soybean meal	11	9	6
Corn	20	20	20
Rice straw	20	20	20
Molasses	4	9	5
Palm kernel oil	3	—	—
Caco_3_	1	1	1
Salt	0.5	0.5	0.5
Mineral and vitamin mix	0.5	0.5	0.5

Chemical composition (% of DM)			
Dry matter	89.79 ± 0.06	89.22 ± 0.03	90.17 ± 0.03
Crude protein	14.25 ± 0.78	14.45 ± 0.35	14.69 ± 0.26
Ether extract	4.86 ± 0.35	5.09 ± 0.10	7.38 ± 0.70
NDF	48.58 ± 1.02	46.63 ± 1.44	48.30 ± 0.72
ADF	30.10 ± 0.34	27.34 ± 0.56	27.09 ± 0.20
Ash	10.19 ± 0.12	9.32 ± 0.16	9.14 ± 0.06
Metabolizable energy (MJ/kg)^2^	11.30	11	11

^1^L0 diet: control diet, containing 0% whole linseed, L10: diet containing 10% whole linseed, and L20: diet containing 20% whole linseed.

^2^Calculate.

**Table 2:** Fatty acid composition of the experimental diets.

Fatty acids	Experimental diets^1^		
	L0	L10	L20
	g/100 g (mean ± SE) of total fatty acids		
C12:0, lauric	5.28^a^ ± 0.87	3.17^b^ ± 0.14	1.97^b^ ± 0.23
C14:0, myristic	2.46^a^ ± 0.33	1.03^b^ ± 0.09	0.31^c^ ± 0.09
C16:0, palmitic	28.07^a^ ± 2.71	9.61^b^ ± 0.20	7.89 ^b^ ± 0.17
C16:1, palmitoleic	0.25 ± 0.05	0.21 ± 0.05	0.17 ± 0.02
C17:0, heptadecanoic	0.72 ± 0.16	0.39 ± 0.09	0.38 ± 0.09
C18:0, stearic	5.92^a^ ± 0.51	3.23^b^ ± 0.67	4.93^a^ ± 0.01
C18:1 n-9, oleic	33.48^a^ ± 1.73	27.67^b^ ± 0.33	27.06^b^ ± 0.1
C18:2 n-6, linoleic	21.88 ± 2.01	21.25 ± 0.55	18.00 ± 0.07
C18:3 n-3, *α*-linolenic	1.92^c^ ± 0.30	33.42^b^ ± 0.23	39.27^a^ ± 0.47

SFA^2^	42.45^a^ ± 3.25	17.44^b^ ± 0.49	15.48^b^ ± 041
UFA^3^	57.55^b^ ± 3.25	82.66^a^ ± 0.49	84.52^a^ ± 0.41
MUFA^4^	33.73^a^ ± 1.92	27.88^b^ ± 0.11	27.23^b^ ± 0.11
PUFA n-3^5^	1.92^c^ ± 0.30	33.42^b^ ± 0.23	39.27^a^ ± 0.47
PUFA n-6^6^	21.88 ± 2.01	21.25 ± 0.55	18.00 ± 0.07
PUFA n-6/n-3	11.40^a ^±^ ^0.72	0.64^b^ ± 0.02	0.46^b^ ± 0.001
UFA/SFA	1.36^c^ ± 0.19	4.74^b^ ± 0.15	5.47^a^ ± 0.02
PUFA/SFA	0.54^c^ ± 0.01	3.14^b^ ± 0.09	3.71^a^ ± 0.01

^1^L0 diet: control diet, containing 0% whole linseed, L10: diet containing 10% whole linseed, and L20: diet containing 20% whole linseed.

^abc^Values with different superscripts within a row differ significantly at *P* < 0.05.

^2^SFA (saturated fatty acids): C12:0 C14:0 + C16:0 + C17:0 + C18:0.

^3^UFA (unsaturated fatty acids): C16:1 + C18:1 n-9 + C18:1n-9 trans + C18:2n-6 + C18:3 n-3.

^4^MUFA (monounsaturated fatty acids): C16:1 + C18:1 n-9.

^5^PUFA n-3: C18:3 n-3.

^6^PUFA n-6: C18:2 n-6.

**Table 3:** Fatty acid profiles of the* longissimus dorsi *muscle of goats fed diets containing different levels of whole linseed.

Fatty acids	Experimental diets^1^		
	L0	L10	L20
	g/100 g (mean ± SE) of total fatty acids		
C12:0, lauric	0.63 ± 0.21	0.54 ± 0.15	0.55 ± 0.08
C14:0, myristic	1.87 ± 0.19	1.75 ± 0.36	1.87 ± 0.32
C15:0, pentadecanoic	0.90 ± 0.06	1.08 ± 0.22	0.90 ± 0.13
C15:1, pentadecenoic	0.77 ± 0.10	0.88 ± 0.16	0.74 ± 0.07
C16:0, palmitic	20.94^a^ ± 0.19	18.11^b^ ± 1.08	18.80^b^ ± 0.93
C16:1, palmitoleic	1.89^a^ ± 0.14	1.37^b^ ± 0.17	1.08^b^ ± 0.13
C17:0, heptadecanoic	1.79 ± 0.24	1.46 ± 0.23	1.31 ± 0.23
C17.1, heptadecenoic	0.66 ± 0.09	0.60 ± 0.05	0.67 ± 0.08
C18:0, stearic	15.09 ± 0.82	15.35 ± 0.72	16.85 ± 1.06
C18:1 n-9, oleic	36.17 ± 1.31	39.45 ± 0.89	36.18 ± 2.04
C18:1 trans-11, vaccenic	1.44 ± 0.15	1.36 ± 0.29	1.99 ± 0.16
C18:2 n-6, linoleic	10.30^a^ ± 1.43	7.03^b^ ± 0.58	6.71^b^ ± 0.78
C18:2 cis-9, trans-11. CLA	0.65 ± 0.19	0.91 ± 0.23	0.77 ± 0.07
C18:2 trans-10, cis-12, CLA	0.47 ± 0.16	0.41 ± 0.08	0.45 ± 0.14
C18:3 n-3, linolenic	0.39^c^ ± 0.09	2.31^b^ ± 0.35	4.85^a^ ± 0.89
C20:4 n-6, arachidonic	4.58^a^ ± 0.38	3.22^b^ ± 0.34	3.22^b^ ± 0.37
C20:5 n-3, eicosapentaenoic	0.22^b^ ± 0.04	1.38^a^ ± 0.24	1.55^a^ ± 0.17
C22:5 n-3, docosapentaenoic	0.50^b^ ± 0.05	1.25^a^ ± 0.27	1.90^a^ ± 0.20
C22:6 n-3, docosahexaenoic	0.63^b^ ± 0.10	0.77^ab^ ± 0.11	1.00^a^ ± 0.12

SFA^2^	41.23 ± 0.51	38.29 ± 1.02	40.17 ± 1.34
UFA^3^	58.77 ± 0.84	61.71 ± 1.02	59.83 ± 1.09
MUFA^4^	40.92 ± 1.35	43.66 ± 0.85	40.67 ± 1.95
PUFA n-3^5^	1.74^c^ ± 0.18	6.36^b^ ± 0.71	9.00^a^ ± 1.06
PUFA n-6^6^	14.88^a^ ± 1.53	10.24^b^ ± 0.90	9.57^b^ ± 1.12
Total CLA^7^	1.11 ± 0.14	1.32 ± 0.24	1.22 ± 0.19
PUFA n-6/n-3	8.86^a^ ± 1.42	1.68^b^ ± 0.17	1.16^b^ ± 0.11
UFA/SFA	1.43^b^ ± 0.03	1.62^a^ ± 0.07	1.50^ab^ ± 0.08
PUFA/SFA	0.40^b^ ± 0.02	0.44^a^ ± 0.04	0.45^a^ ± 0.02

^1^L0 diet: control diet, containing 0% whole linseed, L10: diet containing 10% whole linseed, and L20: diet containing 20% whole linseed.

^abc^Values with different superscripts within a row differ significantly at *P* < 0.05.

^2^SFA (saturated fatty acids): C12:0 + C14:0+ C15:0 + C16:0 + C17:0 + C18:0.

^3^UFA (unsaturated fatty acids): C14:1 + C15:1 + C16:1 + C17:1 + C18:1n-9 + C18:1n-9 trans + C18:2 n-6 + C18:3 n-3 + C20:4 n-6, C20:5n-3 + C22:5 n-3 + C22:6 n-3.

^4^MUFA (monounsaturated fatty acids): C14:1+ C15:1 + C16:1 + C17:1 + C18:1 n-9 + C18:1 n-9 trans.

^5^PUFA n-3: C18:3 n-3 + C20:5 n-3 + C22:5 n-3 + C22:6 n-3.

^6^PUFA n-6 = C18:2 n-6 + C20:4 n-6.

^7^Total CLA: C18:2 cis-9, trans-11 + C18:2 trans-10, cis-12.

**Table 4:** Fatty acid profiles of the *supraspinatus *muscleof goats fed diets containing different levels of whole linseed.

Fatty acids	Experimental diets^1^		
	L0	L10	L20
	g/100 g (mean ± SE) of total fatty acids		
C12:0, lauric	0.31^b^ ± 0.05	0.36^b^ ± 0.05	0.98^a^ ± 0.25
C14:0, myristic	1.73 ± 0.13	1.84 ± 0.19	1.85 ± 0.40
C15:0, pentadecanoic	0.98 ± 0.08	1.20 ± 0.20	1.27 ± 0.22
C15:1, pentadecenoic	0.82 ± 0.06	0.95 ± 0.16	1.10 ± 0.14
C16:0, palmitic	20.55^a^ ± 0.35	18.78^ab^ ± 0.91	16.41^b^ ± 1.21
C16:1, palmitoleic	1.32 ± 0.31	1.47 ± 0.18	1.14 ± 0.19
C17:0, heptadecanoic	1.45 ± 0.20	0.90 ± 0.21	1.87 ± 0.49
C17.1, heptadecenoic	0.59 ± 0.06	0.57 ± 0.06	0.62 ± 0.04
C18:0, stearic	15.66 ± 0.37	15.62 ± 0.82	16.17 ± 0.94
C18:1 n-9, oleic	37.70 ± 1.49	36.35 ± 1.02	32.40 ± 1.43
C18:1 trans-11, vaccenic	0.84^b^ ± 0.23	1.13^ab^ ± 0.13	1.51^a^ ± 0.21
C18:2 n-6, linoleic	10.15 ± 0.87	8.7 ± 0.61	10.4 ± 0.87
C18:2 cis-9, trans-11. CLA	0.72 ± 0.17	0.91 ± 0.25	0.97 ± 0.09
C18:2 trans-10, cis-12, CLA	0.22^b^ ± 0.12	0.65^a^ ± 0.18	0.66^a^ ± 0.15
C18:3 n-3, linolenic	0.36^c^ ± 0.09	2.73^b^ ± 0.31	4.12^a^ ± 0.27
C20:4 n-6, arachidonic	4.7 ± 0.37	3.7 ± 0.39	3.7 ± 0.45
C20:5 n-3, eicosapentaenoic	0.74^b^ ± 0.17	1.27^ab^ ± 0.15	1.76^a^ ± 0.25
C22:5 n-3, docosapentaenoic	0.58^b^ ± 0.10	1.52^a^ ± 0.18	1.63^a^ ± 0.31
C22:6 n-3, docosahexaenoic	0.44^b^ ± 0.05	1.15^a^ ± 0.09	1.22^a^ ± 0.08

SFA^2^	40.70 ± 0.39	38.70 ± 0.96	38.58 ± 0.87
UFA^3^	59.30 ± 0.39	61.30 ± 0.96	61.42 ± 0.87
MUFA^4^	41.27 ± 1.37	40.48 ± 1.01	36.77 ± 1.49
PUFA n-3^5^	2.13^c^ ± 0.26	6.67^b^ ± 0.63	8.74^a^ ± 0.74
PUFA n-6^6^	14.85 ± 1.14	12.37 ± 0.97	14.07 ± 1.29
Total CLA^7^	0.94^b^ ± 0.20	1.56^a^ ± 0.39	1.63^a^ ± 0.19
PUFA n-6/n-3	7.55^a^ ± 1.23	1.87^b^ ± 0.06	1.61^a^ ± 0.06
UFA/SFA	1.46^b^ ± 0.02	1.59^a^ ± 0.06	1.60^a^ ± 0.06
PUFA/SFA	0.42^b^ ± 0.03	0.50^ab^ ± 0.05	0.60^a^ ± 0.06

^1^L0 diet: control diet, containing 0% whole linseed, L10: diet containing 10% whole linseed, and L20: diet containing 20% whole linseed.

^abc^Values with different superscripts within a row differ significantly at *P* < 0.05.

^2^SFA (saturated fatty acids): C12:0 C14:0 + C15:0 + C16:0 + C17:0 + C18:0.

^3^UFA (unsaturated fatty acids): C14:1 + C15:1 + C16:1 + C17:1 + C18:1n-9 + C18:1n-9 trans + C18:2 n-6 + C18:3 n-3 + C20:4 n-6, C20:5 n-3 + C22:5 n-3 + C22:6 n-3.

^4^MUFA (monounsaturated fatty acids): C14:1 + C15:1 + C16:1 + C17:1 + C18:1 n-9 + C18:1 n-9 trans.

^5^PUFA n-3: C18:3 n-3 + C20:5 n-3 + C22:5n-3 + C22:6 n-3.

^6^PUFA n-6: C18:2 n-6 + C20:4 n-6.

^7^Total CLA: C18:2 cis-9, trans-11+ C18:2 trans-10, cis-12.

**Table 5:** Fatty acid profiles ofthe *semitendinosus *muscle of goats fed diets containing different levels of whole linseed.

Fatty acids	Experimental diets		
	L0	L10	L20
	g/100 g (mean ± SE) of total fatty acids		
C12:0, lauric	0.36 ± 0.07	0.28 ± 0.08	0.51 ± 0.12
C14:0, myristic	2.64 ± 0.38	2.43 ± 0.49	2.12 ± 0.26
C15:0, pentadecanoic	0.48^b^ ± 0.03	0.93^a^ ± 0.11	0.81^a^ ± 0.06
C15:1, pentadecenoic	0.41^b^ ± 0.03	0.75^a^ ± 0.09	0.66^a^ ± 0.05
C16:0, palmitic	24.99^a^ ± 1.10	19.58^b^ ± 0.57	18.98^b^ ± 0.58
C16:1, palmitoleic	2.19 ± 0.46	1.96 ± 0.42	1.40 ± 0.15
C17:0, heptadecanoic	1.41 ± 0.36	2.06 ± 0.54	2.69 ± 0.52
C17.1, heptadecenoic	0.57 ± 0.04	0.64 ± 0.09	0.56 ± 0.05
C18:0, stearic	16.02 ± 0.31	15.46 ± 0.99	16.43 ± 0.93
C18:1 n-9 cis, oleic	38.03^a^ ± 0.41	38.09^a^ ± 0.90	35.32^b^ ± 0.52
C18:1 trans-11, vaccenic	1.65 ± 0.11	1.31 ± 0.13	1.41 ± 0.20
C18:2 n-6, linoleic	6.28 ± 0.64	6.99 ± 0.53	7.22 ± 0.51
C18:2 cis-9, trans-11. CLA	0.39^c^ ± 0.04	0.79^b^ ± 0.04	1.04^a^ ± 0.13
C18:2 trans-10, cis-12, CLA	0.15 ± 0.11	0.49 ± 0.27	0.23 ± 0.07
C18:3 n-3, linolenic	0.30^c^ ± 0.10	2.12^b^ ± 0.12	3.47^a^ ± 0.33
C20:4 n-6, arachidonic	3.02 ± 0.68	3.17 ± 0.20	2.82 ± 0.19
C20:5 n-3, eicosapentaenoic	0.51^c^ ± 0.02	1.01^b^ ± 0.04	1.54^a^ ± 0.12
C22:5 n-3, docosapentaenoic	0.32^c^ ± 0.02	0.91^b^ ± 0.07	1.14^a^ ± 0.11
C22:6 n-3, docosahexaenoic	0.3^b^ ± 0.08	0.9^a^ ± 0.12	1.1^a^ ± 0.19

SFA^2^	45.88^a^ ± 1.15	40.74^b^ ± 0.53	41.54^b^ ± 0.62
UFA^3^	54.12^b^ ± 1.15	59.26^a^ ± 0.53	58.56^a^ ± 0.62
MUFA^4^	42.85^a^ ± 0.50	42.75^a^ ± 1.00	39.36^b^ ± 0.64
PUFA n-3^5^	1.40^c^ ± 0.17	5.07^b^ ± 0.13	7.67^a^ ± 0.48
PUFA n-6^6^	9.32 ± 1.31	10.16 ± 0.69	10.03 ± 0.59
Total CLA^7^	0.54^b^ ± 0.07	1.27^a^ ± 0.29	1.27^a^ ± 0.17
PUFA n-6/n-3	6.65^a^ ± 0.48	2.00^b^ ± 0.09	1.32^c^ ± 0.06
UFA/SFA	1.18^b^ ± 0.06	1.46^a^ ± 0.03	1.41^a^ ± 0.03
PUFA/SFA	0.24^b^ ± 0.04	0.37^a^ ± 0.02	0.43^a^ ± 0.03

^1^L0 diet: control diet, containing 0% whole linseed, L10: diet containing 10% whole linseed, and L20: diet containing 20% whole linseed.

^abc^Values with different superscripts within a row differ significantly at *P* < 0.05.

^2^SFA (saturated fatty acids): C12:0 C14:0 + C15:0 + C16:0 + C17:0 + C18:0.

^3^UFA (unsaturated fatty acids): C14:1 + C15:1 + C16:1 + C17:1 + C18:1n-9 + C18:1n-9 trans + C18:2 n-6 + C18:3 n-3 + C20:4 n-6, C20:5 n-3 + C22:5 n-3 + C22:6 n-3.

^4^MUFA (monounsaturated fatty acids): C14:1 + C15:1 + C16:1 + C17:1 + C18:1 n-9 + C18:1 n-9 trans.

^5^PUFA n-3: C18:3 n-3 + C20:5 n-3 + C22:5n-3 + C22:6 n-3.

^6^PUFA n-6: C18:2 n-6 + C20:4 n-6.

^7^Total CLA: C18:2 cis-9, trans-11 + C18:2 trans-10, cis-12.

**Table 6:** Fatty acid profiles of the* subcutaneous *fat of goats fed diets containing different levels of whole linseed.

Fatty acids	Experimental diets^1^		
	L0	L10	L20
	g/100 g (mean ± SE) of total fatty acids		
C12:0, lauric	0.55 ± 0.26	0.11 ± 0.02	0.31 ± 0.09
C14:0, myristic	0.38 ± 0.18	0.11 ± 0.01	0.32 ± 0.08
C15:0, pentadecanoic	1.38 ± 0.42	0.75 ± 0.04	0.86 ± 0.18
C15:1, pentadecenoic	0.69 ± 0.26	0.21 ± 0.01	0.35 ± 0.14
C16:0, palmitic	23.77^a^ ± 1.67	22.80^b^ ± 0.56	21.41^b^ ± 0.71
C16:1, palmitoleic	0.50^b^ ± 0.20	0.94^b^ ± 0.25	1.82^a^ ± 0.19
C17:0, heptadecanoic	1.51^b^ ± 0.21	0.63^b^ ± 0.17	1.06^ab^ ± 0.14
C17.1, heptadecenoic	1.65 ± 0.34	1.40^a^ ± 0.12	1.77 ± 0.15
C18:0, stearic	26.76^a^ ± 2.50	23.72^b^ ± 1.07	21.44^b^ ± 3.47
C18:1 n-9 cis, oleic	37.14^b^ ± 1.16	43.13^a^ ± 0.67	39.90^b^ ± 1.43
C18:1 trans-11, vaccenic	1.65^b^ ± 0.53	1.72^b^ ± 0.25	4.96^a^ ± 0.73
C18:2 n-6, linoleic	2.80 ± 0.91	2.20 ± 0.25	2.88 ± 1.26
C18:2 cis-9, trans-11. CLA	0.64^b^ ± 0.34	0.68^b^ ± 0.25	1.23^a^ ± 0.19
C18:2 trans-10, cis-12, CLA	0.24 ± 0.05	0.35 ± 0.06	0.30 ± 0.07
C18:3 n-3, linolenic	0.28^c^ ± 0.06	0.86^b^ ± 0.13	1.94^a^ ± 0.26
C20:4 n-6, arachidonic	0.15^b^ ± 0.05	0.47^a^ ± 0.10	0.33^ab^ ± 0.08

SFA^2^	53.98^a^ ± 1.06	48.01^b^ ± 0.91	45.08^b^ ± 2.88
UFA^3^	46.02^b^ ± 1.06	51.99^a^ ± 0.91	54.92^b^ ± 2.88
MUFA^4^	42.01^b^ ± 1.06	42.93^a^ ± 0.74	45.41^ab^ ± 2.20
PUFA n-3^5^	0.28^c^ ± 0.06	0.86^b^ ± 0.13	1.94^a^ ± 0.26
PUFA n-6^6^	2.95 ± 0.93	2.67 ± 0.17	3.20 ± 1.21
Total CLA^7^	0.88^b^ ± 0.36	1.04^ab^ ± 0.20	1.53^a^ ± 0.17
PUFA n-6/n-3	15.79^a^ ± 1.90	3.67^b^ ± 0.53	1.60^c^ ± 0.63
UFA/SFA	0.85^b^ ± 0.04	1.08^a^ ± 0.05	1.22^a^ ± 0.14
PUFA/SFA	0.06^b^ ± 0.02	0.08^b^ ± 0.01	0.11^a^ ± 0.04

^1^L0 diet: control diet, containing 0% whole linseed, L10: diet containing 10% whole linseed, and L20: diet containing 20% whole linseed.

^abc^Values with different superscripts within a row differ significantly at *P* < 0.05.

^2^SFA (saturated fatty acids): C12:0 + C14:0 + C15:0 + C16:0 + C17:0 + C18:0.

^3^UFA (unsaturated fatty acids): C15:1 + C16:1 + C17:1 + C18:1n-9 + C18:1n-9 trans + C18:2 n-6 + C18:3 n-3 + C20:4 n-6.

^4^MUFA (monounsaturated fatty acids): C14:1+ C15:1 + C16:1 + C17:1 + C18:1 n-9 + C18:1 n-9 trans.

^5^PUFA n-3: C18:3 n-3.

^6^PUFA n-6: C18:2 n-6 + C20:4 n-6.

^7^Total CLA: C18:2 cis-9, trans-11 + C18:2 trans-10, cis-12.

**Table 7:** Fatty acid profiles of perirenal fat of goats fed diets containing different levels of whole linseed.

Fatty acids	Experimental diets^1^		
	L0	L10	L20
	g/100 g (mean ± SE) of total fatty acids		
C12:0, lauric	0.40 ± 0.11	0.38 ± 0.07	0.38 ± 0.07
C14:0, myristic	0.38 ± 0.10	0.39 ± 0.09	0.34 ± 0.09
C15:0, pentadecanoic	0.59 ± 0.01	0.64 ± 0.02	0.66 ± 0.03
C15:1, pentadecenoic	0.21 ± 0.04	0.34 ± 0.09	0.30 ± 0.06
C16:0, palmitic	33.83^a^ ± 0.60	26.90^b^ ± 0.97	23.65^c^ ± 1.12
C16:1, palmitoleic	1.33 ± 0.12	1.02 ± 0.14	0.98 ± 0.13
C17:0, heptadecanoic	1.07 ± 0.07	1.09 ± 0.04	1.24 ± 0.09
C17.1, heptadecenoic	0.31 ± 0.01	0.57 ± 0.10	0.54 ± 0.12
C18:0, stearic	33.14 ± 0.87	37.93 ± 3.03	37.78 ± 2.59
C18:1 n-9 cis, oleic	23.66 ± 0.76	24.40 ± 2.50	25.08 ± 3.16
C18:1 trans-11, vaccenic	2.07 ± 0.07	1.84 ± 0.24	1.41 ± 0.30
C18:2 n-6, linoleic	1.81^b^ ± 0.13	2.0^b^ ± 0.30	3.74^a^ ± 0.49
C18:2 cis-9, trans-11. CLA	0.40^c^ ± 0.05	0.75^b^ ± 0.07	0.95^c^ ± 0.12
C18:2 trans-10, cis-12, CLA	0.25 ± 0.06	0.27 ± 0.08	0.30 ± 0.04
C18:3 n-3, linolenic	0.22^c^ ± 0.03	0.66^b^ ± 0.08	1.67^a^ ± 0.27
C20:4 n-6, arachidonic	0.48 ± 0.08	0.50 ± 0.11	0.43 ± 0.09

SFA^2^	69.03^a^ ± 0.90	66.94^ab^ ± 2.53	63.71^a^ ± 3.13
UFA^3^	30.97 ± 0.90	33.06 ± 2.53	36.29 ± 3.13
MUFA^4^	27.95 ± 0.84	28.56 ± 2.52	28.64 ± 3.11
PUFA n-3^5^	0.22^c^ ± 0.03	0.66^b^ ± 0.08	1.67^a^ ± 0.27
PUFA n-6^6^	2.61^b^ ± 0.13	2.50^b^ ± 0.30	4.17^a^ ± 0.49
Total CLA^7^	0.64^c^ ± 0.10	1.02^b^ ± 0.10	1.25^a^ ± 0.10
PUFA n-6/n-3	10.53^a^ ± 1.33	3.14^b^ ± 0.31	2.72^b^ ± 0.29
UFA/SFA	0.45 ± 0.02	0.51 ± 0.07	0.59 ± 0.10
PUFA/SFA	0.03^b^ ± 0.002	0.05^b^ ± 0.004	0.10^a^ ± 0.01

^1^L0 diet: control diet, containing 0% whole linseed, L10: diet containing 10% whole linseed, and L20: diet containing 20% whole linseed.

^abc^Values with different superscripts within a row differ significantly at *P* < 0.05.

^2^SFA (saturated fatty acids): C12:0 C14:0 + C15:0 + C16:0 + C17:0 + C18:0.

^3^UFA (unsaturated fatty acids): C15:1 + C16:1 + C17:1 + C18:1n-9 + C18:1n-9 trans + C18:2 n-6 + C18:3 n-3 + C20:4 n-6.

^4^MUFA (monounsaturated fatty acids): C15:1 + C16:1 + C17:1 + C18:1 n-9 + C18:1 n-9 trans.

^5^PUFA n-3: C18:3 n-3.

^6^PUFA n-6: C18:2 n-6 + C20:4 n-6.

^7^Total CLA: C18:2 cis-9, trans-11 + C18:2 trans-10, cis-12.
